# Congenital Myasthenic Syndrome Caused by a Novel Hemizygous *CHAT* Mutation

**DOI:** 10.3389/fped.2020.00185

**Published:** 2020-04-28

**Authors:** Yixia Zhang, Xinru Cheng, Chenghan Luo, Mengyuan Lei, Fengxia Mao, Zanyang Shi, Wenjun Cao, Jingdi Zhang, Qian Zhang

**Affiliations:** Neonatal Intensive Care Unit, The First Affiliated Hospital of Zhengzhou University, Zhengzhou, China

**Keywords:** congenital myasthenic syndrome, CHAT mutation, hemizygous, apnoea, genetic diagnosis

## Abstract

Congenital myasthenic syndrome (CMS) is a neuromuscular transmission disorder caused by mutations in genes encoding neuromuscular junction proteins. CMS due to choline acetyltransferase (*CHAT*) gene mutation is characterized by episodic apnoea. To date, 52 cases of CMS caused by *CHAT* gene mutations have been reported. Here, we report a neonate with the third hemizygous mutation [a 4.9 Mb deletion [10q11.22–10q11.23 (chr10: 46123781–51028772)] containing the whole *CHAT* gene and c.1976A>T (p.Gln659Leu in the *CHAT* gene)]. The c.1976A>T (p.Gln659Leu) variant had not been reported in the ExAC or gnomAD databases and was predicted to be pathogenic. The alignment of amino acid sequences revealed that glutamine at codon 659 is highly conserved in different species and causes structural changes in the substrate-binding site. Our female patient with neonate-onset CMS presented with apnoea, dyspnoea, feeding difficulties, weak crying, and seizure-like episodes, and her respiration was ventilator dependent. The prostigmine test was positive. This case may help to further elucidate clinical features and treatment methods in neonate-onset CMS caused by *CHAT* gene mutations.

## Introduction

Congenital myasthenic syndromes (CMSs) are rare genetically heterogeneous diseases caused by abnormal signal transduction due to mutations in genes coding for proteins expressed at the neuromuscular junction (NMJ). According to the site of mutation, CMS can be divided into presynaptic, synaptic or postsynaptic (1). Individuals with presynaptic types commonly present with apnoea and can also present with dyspnoea, ventilator dependence, ptosis, bulbar paralysis, and fatigue. The above symptoms may be triggered by fever, cold, stress, infection, excitement, and excessive exercise and may lead to apnoea crisis. Arthrogryposis can also be seen in presynaptic types of CMS. Individuals with synaptic types can present with delayed pupillary light reflex; renal and ocular malformations; Pierson syndrome; and severe ocular, respiratory, and proximal limb muscle weakness. Patients typically present with apnoea and generalized weakness in the neonatal period or infancy that persists throughout life. Individuals with postsynaptic types can present with ptosis and ophthalmoparesis; micrognathia; lesser face symptoms; and weakness of the masticatory, limb girdle and neck muscles in the first year of life, and arthrogryposis at birth occurs in close to one-third of patients. Patients presenting later in childhood often have a milder clinical course. A useful clinical clue is selectively severe involvement of cervical, wrist, and extensor muscles (1–3). CMSs, with the exception of slow channel syndrome, SNAP25-CMS and synaptotagmin 2–CMS, are due to autosomal recessive hereditary mutations (4–6). Several novel CMS mutations have been discovered since 2011 due to the development of whole exome sequencing. Protein glycosylation with enzymatic defects, that is, posttranslational addition of glucose or mannose residues that occurs in the Golgi apparatus and endoplasmic reticulum, has been identified as a new subtype of CMS (7). To date, more than 30 CMS disease genes have been identified (8, 9).

*CHAT* gene mutations are presynaptic types. They account for approximately 5% of CMS probands (1). The *CHAT* gene is located on the long arm of human chromosome 10 (10q11.23) and encodes the biosynthetic enzyme of the neurotransmitter acetylcholine, namely, choline acetyltransferase. *CHAT* gene mutations can result in deficient resynthesis of acetylcholine following its reuptake at the nerve terminal (10). The choline acetyltransferase protein belongs to the family of eukaryotic acetyltransferases (11). To date, 48 *CHAT* gene mutations have been identified as related to CMS (http://www.hgmd.cf.ac.uk/ac/dipx.php). Mutations located near the active-site tunnel, impairing substrate binding, result in more severe phenotypic effects in patients with *CHAT*-related CMS (12–14). Fifty-two cases of CMS caused by *CHAT* gene mutations have been reported, and only two of them have been identified as hemizygous mutations (12, 13, 15–29). In 2011, Shen et al. revealed the first hemizygous *CHAT* mutation in a patient with CMS by quantitative RT-PCR (12). Schwartz et al. reported the second hemizygous *CHAT* mutation in a patient with CMS in 2018 (16).

Here, we report a third case of hemizygous mutation. A neonate with CMS carried a 4.9 Mb deletion and a c.1976A>T (p.Gln659Leu) mutation in the *CHAT* gene. The deleted region, q11.22–q11.23 (chr10: 46123781-51028772) × 1, included 91 genes. Only five were associated with abnormal phenotypes in the Online Mendelian Inheritance in Man (OMIM®) database: *CHAT* (choline acetyltransferase), *ERCC6* (chromatin remodeling factor), *SLC18A3* (solute carrier family 18, member 3), *GDF2* (growth differentiation factor 2), and *RBP3* (retinol binding protein 3). Whole exome sequencing showed a hemizygous mutation in *CHAT* (c.1976A>T, p.Gln659Leu), which likely accounts for the patient's phenotype. The clinical features and treatment of this neonate were discussed below.

## Case Report

### Medical history

The proband was a 13-day-old female, the second child of healthy non-consanguineous parents. She was born as the first of fraternal twins at 37 weeks via cesarean section due to reduced heart rate of the twins. Her birth weight was 2.4 kg (<10th p). Her older brother and the other neonate twin were both normal, and there was no relevant medical family history. After birth, she required a laryngeal mask to pressurize the oxygen. The treatment relieved her cyanosis. She was then immediately transferred to the local neonatal intensive care unit where she was intubated and ventilated. She was also treated with anti-infectious and excitatory respiration center drugs (nalmefene hydrochloride injection). However, the effect was poor. She failed to extubate after multiple attempts due to carbon dioxide retention. The patient was hospitalized in our neonatal intensive care unit for dyspnoea for 13 days with failure to extubate for 1 day.

### Physical Examination

The patient had a full-term appearance, a weight of 2.35 kg, poor response to physical examinations, weak crying, labored breathing, shortness of breath, and a positive three-concave sign. Both lungs were clear on auditory percussion. Neither obvious wet nor dry rattling was heard. The heart rate was strong, and no obvious murmurs were heard in the precordial region. There was no abdominal guarding upon palpation, and abdominal rumbling was weak. There was reduced movement of the limbs, decreased muscle tone and absent primitive reflexes.

### Main Laboratory Examinations

**Figure 1:**
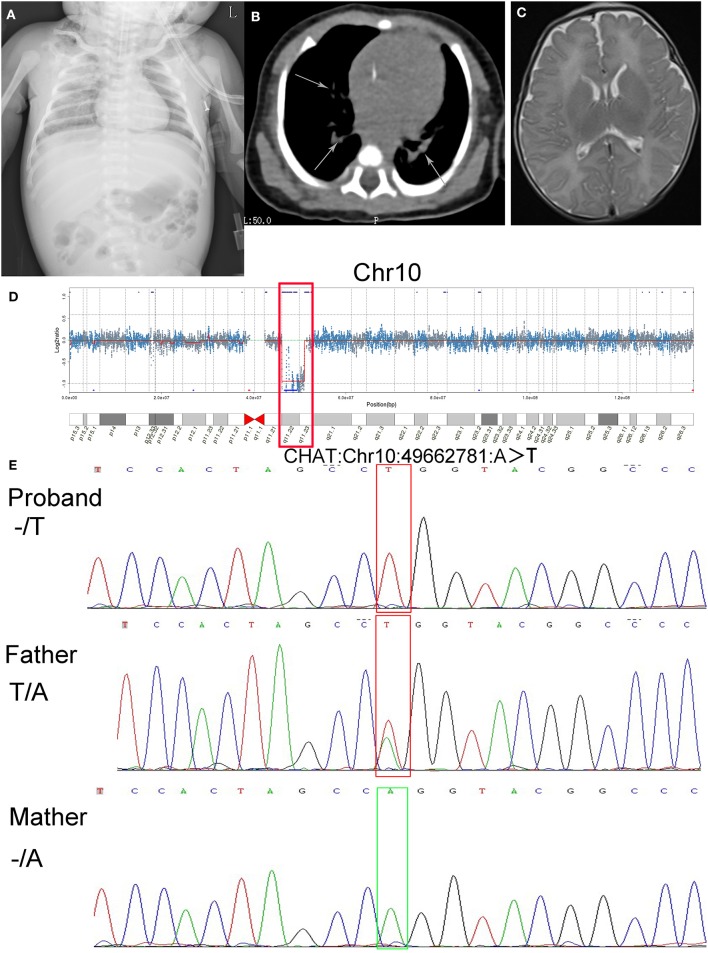
Results of imaging tests and genetic tests, alignment of amino acid sequences, and a schematic of gene structure and protein structure. **(A)** Chest plain film indicated double-lung texture growing thick and heavy. **(B)** CT images showed an increasing density of patchy shadows with blurry margins (arrows). **(C)** The brain MRI showed no abnormalities. **(D)** The results of CNV-Seq from the patient's genomic DNA sample (q11.22–q11.23 deletion on chromosome 10 is shown in the box). **(E)** Sanger sequencing of the CHAT gene showed the hemizygous mutation c.1976A>T (p.Gln659Leu) in the proband, absence of the mutation in the mother and heterozygous mutation in the father.

The results of routine blood examination were as follows: white blood cell count 27.90 × 10^9^/L, erythrocyte count 3.61 × 10^12^/L, hemoglobin 113.0 g/L, platelet count 390 × 10^9^/L, percentage of neutrophils 55.8%, and percentage of lymphocytes 32.8%. The levels of inflammatory markers were as follows: procalcitonin 0.390 ng/mL and C-reactive protein 7.60 mg/L. The N-terminal pro b-type natriuretic peptide level was 1,334.00 pg/mL. The prostigmine test was positive. The fungal glucan level was 405.05 pg/mL. The myocardial enzyme levels were as follows: creatine kinase 70.00 U/L and creatine kinase isoenzyme 20.10 U/L. The chest plain film indicated a double-lung texture growing thick and heavy, and CT images showed an increasing density of patchy shadows with blurry margins, which was consistent with the manifestations of pneumonia (Figures 1A,B). No abnormalities were found on cranial MRI (Figure 1C). In regard to brain function, the upper and lower peaks were generally normal, but the differences in the sleep-wake cycle were not obvious. The acetylcholine receptor antibody level was 0.045 mmol/L (within the normal range).

### Therapeutic Process

Upon transfer to our neonatal intensive care unit, the patient's oxygen saturation decreased frequently and at times was accompanied by convulsions. Subsequently, the patient was intubated for mechanical ventilation, was fed milk through a nasogastric tube and later showed difficulty in weaning from the ventilator. We administered a neostigmine (0.05 mg/kg) intramuscular injection, which relieved the dyspnoea and improved her feeding. We then orally administered pyridostigmine (5 mg/kg) to replace the intramuscular injection, which controlled her dyspnoea. We did not observe any side effects from these drugs. Later, she was weaned successfully from mechanical ventilation and could gradually breathe independent of oxygen therapy. However, episodes of dyspnoea accompanied by cyanosis and decreased oxygen saturation occurred upon reducing the dosage of pyridostigmine. We failed to make a definitive diagnosis at the initial stage of treatment due to rejection of genetic testing by the family members. She was transferred to a hospital with better equipment and treatment capacity, according to the parent's wishes, where she died.

### Genetic Tests

In consideration of the clinical course of the neonate, we suspected that she had CMS. To explore the potential genetic etiology, we communicated with the patient's parents repeatedly and ultimately obtained informed consent for genetic tests 15 days after admission. The genetic testing was performed when she was 28 days old (2018-05-14). Two milliliters of peripheral blood was extracted from the proband and her parents. The blood was sent to Cipher Gene LLC, and the following tests were conducted. Whole exome sequencing was performed on the proband and her parents using the Illumina NovaSeq 6000 platform. Agilent SSELXT Human All Exon V6 was used to capture and construct the library, and a paired-end sequencing strategy was used. CNV-Seq was performed on the proband using the Illumina NovaSeq 6000. A TruSeq Library Construction Kit was used to capture and construct the library, and a paired-end sequencing strategy was used. CNV-Seq indicated a 4.9 Mb deletion [10q11.22-10q11.23 (chr10: 46123781-51028772)], which contained the whole *CHAT* gene (Figure 1D). The whole-exome sequencing validated by Sanger sequencing indicated that the patient carries a hemizygous mutation in exon14 of *CHAT* (c.1976A>T, p.Gln659Leu), which is inherited from her father (Figure 1E).

## Discussion

**Figure 2:**
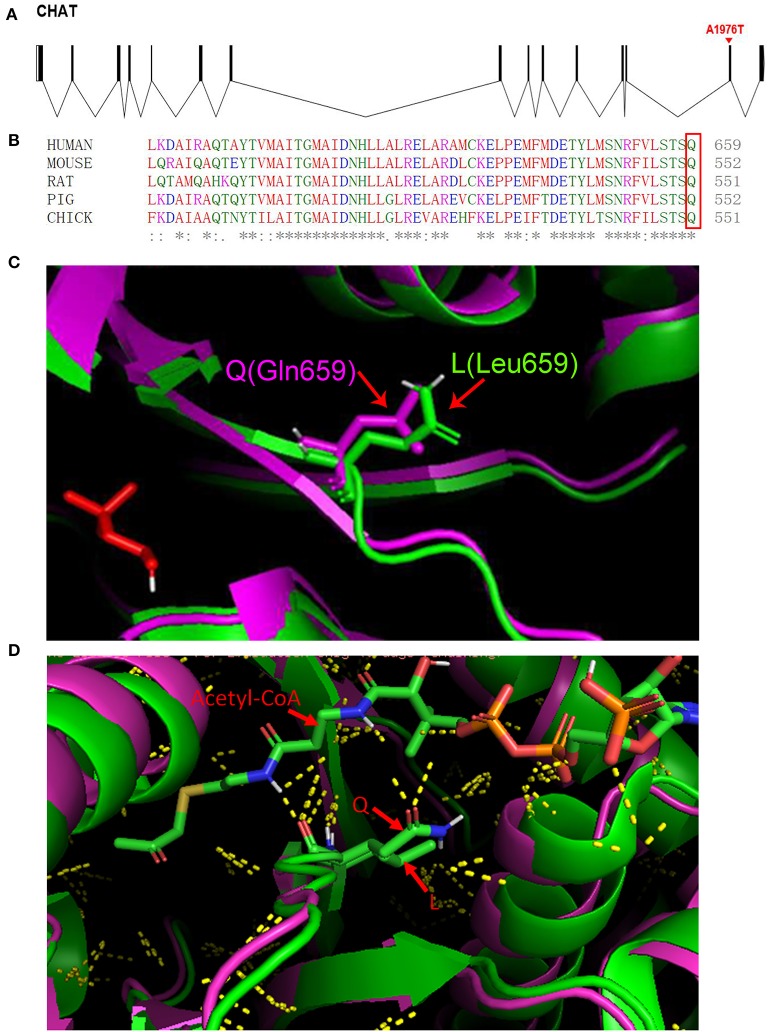
**(A)** The c.1976A>T (p.Gln659Leu) mutation is shown in a schematic of the gene structure. **(B)** (Q) Glutamine at position 659 is highly conserved in CHAT in various species (the box marks the amino acid of interest). **(C)** Homology models for *CHAT* protein generated using the crystal structures of *CHAT* protein (PDB accession code: 2FY4) as the template. **(D)** Cartoon structure representation of the subtle changes (Gln-to-Leu) at position 659. The green cartoon represents the wild-type protein, and the purple cartoon represents the mutant protein. Yellow dots represent polar contacts.

Our patient presented with dyspnoea, ventilator dependence, and apnoea. The prostigmine test was positive. Pyridostigmine relieved dyspnoea in our patient, and she weaned successfully from mechanical ventilation and could gradually breathe independent of oxygen therapy. The clinical course of the neonate and whole exome sequencing indicated *CHAT*-related CMS.

fifty-two cases of *CHAT*-related CMS have been reported to date (Table S1), and our literature search revealed a total of 48 genetic variations that have been identified to date. Our patient carried a 4.9 Mb deletion [10q11.22-10q11.23 (chr10: 46123781-51028772)] and a hemizygous mutation in *CHAT* (p.Gln659Leu). The copy number variation of the 4.9 Mb deletion in this study and its highly overlapping deletion fragment could not be found in the Genetic Variation Database. Highly overlapping pathogenic or suspected pathogenic CNVs were found in the Decipher patient database (https://decipher.sanger.ac.uk/syndromes#syndromes/overview (Table S2). The mutation of p.Gln659Leu was located on the 14 exon of *CHAT* (Figure 2A). The variant had not been reported in the ExAC (http://exac.broadinstitute.org/) or gnomAD databases (http://gnomad.broadinstitute.org/about) and was predicted to be pathogenic by multiple prediction software programs (SIFT, Polyphen2, LRT, Mutation Taster, FATHMM, etc.). The alignment of amino acid sequences revealed that glutamine at codon 659 is highly conserved in different species (https://www.ebi.ac.uk/Tools/msa/), which suggests p.Gln659Leu is likely pathogenic (Figure 2B). Three-dimensional structures of the wild-type and mutant *CHAT* protein of human were constructed by the homologous modeling database SWISS-MODEL (http://swissmodel.expasy.org/). Residual changes between the wild-type and mutant *CHAT* proteins were mapped to the model by PyMol v1.3 software. This mutation causes a structural change in the substrate-binding site (Figures 2C,D). Mutations located near the active-site tunnel impair substrate binding and result in more severe phenotypic effects in patients with *CHAT*-related CMS (12–14). Glutamine possesses features of polarity compared with non-polar leucine due to the amide group in the residue side chain. As shown in Figure 2D, higher numbers of polar contacts between Q (amide group) and acetyl coenzyme A (amino group in the alanine residue and hydroxyl group in diphosphate) might indicate stronger substrate binding in the wild type. However, the hydrophobic alkyl group in the leucine residue side chain might impair substrate binding. Therefore, we speculate the mutation reduces catalytic activity of the enzyme resulting in the defect of neuromuscular transmission.

The current treatment for CMS is dependent on the CMS gene involved. The treatments of *CHAT*-associated CMS are mainly cholinesterase inhibitors and respiratory support, which can help improve clinical symptoms and prognosis. The most commonly used cholinesterase inhibitor is pyridostigmine, which can be used for the treatment of *CHAT*-related CMS pediatric patients. Apnoeic episodes can be relieved with oral or parenteral neostigmine administration (30). In our patient, apnoea and dyspnoea were significantly improved with intramuscular injection of neostigmine, and she was weaned from the ventilator successfully after pyridostigmine (4 mg/kg/day) administration. After 14 days of treatment, we reduced the dosage of pyridostigmine (2 mg/kg/day); however, the oxygen saturation was reduced. We considered that the crisis may have been caused by uncontrolled infection, insufficient drug administration or rapid drug reduction. Patients with *CHAT*-related CMS may have symptoms accompanied by convulsions, which may be easily mistaken for epileptic seizures (27). Our patient's symptoms were initially accompanied by convulsions. Sedative drugs with bolus injection had poor anticonvulsive effects in our case, a finding that warrants attention. It is important to carefully select drug therapy because the same drug can be effective, invalid, or even harmful for different CMSs. For instance, the use of cholinesterase inhibitor in CMS caused by collagen-like tail subunit of acetylcholinesterase mutation is invalid and worsens the condition by enhancing the effect of cholinesterase (30). Patients diagnosed with CMS are rare, and clinical trials to determine optimal pharmacotherapeutic combinations for patients remain difficult to perform. Therefore, a standard treatment for patients with *CHAT* mutations has not been established.

We suggest that all patients suspected of having CMS receive precise genetic diagnosis as soon as possible. We report here a case of CMS caused by a novel hemizygous mutation of the *CHAT* gene and hope this report helps improve physicians' knowledge of *CHAT*-related CMS.

## Data Availability Statement

Publicly available datasets were analyzed in this study. This data can be found here: http://gnomad.broadinstitute.org/about.

## Ethics Statement

We obtained written informed consent to publish this case. This study was approved by the First Affiliated Hospital of Zhengzhou University ethics committee (ID No: 2019-LW-010).

## Author Contributions

QZ conceived and designed the work. YZ drafted the manuscript and collected the data. XC contributed to the conception of the work and played an important role in interpreting the clinical data. CL contributed to the manuscript revision. ML contributed to figure preparation. FM contributed to the adjustment of the structure of the manuscript. ZS contributed to figure preparation. WC contributed to table preparation. JZ contributed to data collection.

## Conflict of Interest

The authors declare that the research was conducted in the absence of any commercial or financial relationships that could be construed as a potential conflict of interest.
