# Supplementary material for: Congenital Myasthenic Syndrome Caused by a Novel Hemizygous CHAT Mutation
Source: Front Pediatr. 2020 Apr 28;8:185. doi: 10.3389/fped.2020.00185 (PMC7198756; doi:10.3389/fped.2020.00185)
Supplement: Supplementary file 1 [file Table_1.DOCX]

Table S1．Clinical features of CMS patients caused by *CHAT* mutations.

| Origin/ case number | Age at onset | main manifestations | Therapy | prognosis | Reference |
| --- | --- | --- | --- | --- | --- |
| Britain  5 | N-early infancy | [apnoea](C:/Users/zhang/AppData/Local/Youdao/dict/Application/8.8.0.0/resultui/html/index.html#/javascript:;), myasthenia, Bp,  increased weakness | U | No abnormal in neurodevelopment | Ohn et al.  [1]  2001 |
| America  1 | N | apnoea, ptosis, fatigued,  speech became slurred after crying or vomiting | P/+ | Motor development delay | Byring et al.  [2]  2002 |
| America  5 | N-18 Mo | apnoea, ptosis, Rd,  frequent falls | P/+,- | No abnormal in neurodevelopment, No abnormal in motor development | Maselli et al.  [3]  2003 |
| Croatia  2 | N | Rd, fatigued, ptosis, hypotonia, apnoea often occurred in the prone position, irregular weak and shallow respiration | P/+ | Motor development delay and speech development delayed(1),  No abnormal in neurodevelopment and No abnormal in motor development (1) | Barisic et al.  [4]  2005 |
| America  1 | N | apnoea, ptosis, severe gastroesophageal reflux | P,E/+ | Motor development delay | Mallory et al.  [5]  2009 |
| Turkey  Finland  Germany  Italy  Lebanon  11 | N-2 y | Neonate-onset: Bp, Vd, severe myasthenia  Infant-onset: Slight persistent myasthenia  apnoea, hypotonia, ptosis in both groups | CI/+ | Died(1), Ndd (9)，  motor development delay (8)  No abnormal in neurodevelopment and No abnormal in motor development (1) | Schara et al.  [6]  2010 and  Yis et al.  [7]  2017 |
| U  11 | N-early infancy | apnoea, Rd, dyspnea, fatigued, hypotonia, Ps, paucity of anti-gravity | P/+(6),-(4) | Died(1), Vd(3)  Multiple arthrogryposis(1) | Shen et al.  [8]  2011 and  Yeung et al.  [9]  2009 |
| America Vietnam  4 | N-18 Mo | Rd, ptosis, Bp, dyspnea, limb movements, diplopia | CI/+ | Motor development delay (4) | Arredondo et al. [10]  2015 |
| Malaysia 2 | U | Motor weakness, ptosis | P/+ | Motor development delay (2) | Tan et al.  [11]  2016 |
| Spain  2 | N-4 y | Rd, myasthenia, ptosis, Ps, dyspnea | U | U | Natera-De et al.  [12]  2017 |
| France  1 | N | ptosis, Da fm, Rd seizure-like episodes, opthalmoplegia nasal gastro-esophageal reflux | P/+ | A slight motor development delay | Schwartz et al.  [13]  2018 |
| China  2 | N-3 Mo | apnoea, dyspnea, ptosis, Feeding difficulties, Weak crying, milk choking | P/+ | Died(1)  Neurodevelopment delay (1) | Liu et al.  [14]  2018 |
| Britain  3 | N-1 y | apnoea, hypotonia, fatigued, ptosis,  Da fm | CI+ | U | Mcmacken et al.  [15]  2018 |
| Turkey  1 | N | apnoea, hypotonia, perioral cyanosis,  loss of consciousness, ptosis, muscle weakness | P/+ | Normal psychomotor development(During 18 months follow-up period) | Arican et al.  [16]  2018 |
| China  1 | N | apnoea, ptosis, weak crying, feeding difficulties | - | Died | Liu et al.  [17]  2019 |

Abbreviations: U=unknown; N=neonate; Mo=month; y=year(s); Bp=bulbar paralysis; Rd=respiratory distress; Ps= poor sucking; Vd=ventilator dependent; Da fm=decreased active fetal movements; P= pyridostigmine; +=effective; -=no effective; E=edrophonium; CI=cholinesterase inhibitors.

**References**

1. Ohno K, Tsujino A, Brengman JM, Harper CM, Bajzer Z, Udd B, et al. Choline Acetyltransferase Mutations Cause Myasthenic Syndrome Associated with Episodic Apnea in Humans. Proceedings of the National Academy of Sciences of the United States of America. 2001;98(4):2017-2022.

2. Byring RF, Pihko H, Tsujino A, Shen XM, Gustafsson B, Hackman P, et al. Congenital myasthenic syndrome associated with episodic apnea and sudden infant death. Neuromuscular Disorders. 2002;12(6):548-553.

3. Maselli RA, Chen D, Mo D, Bowe C, Fenton G, Wollmann RL. Choline acetyltransferase mutations in myasthenic syndrome due to deficient acetylcholine resynthesis. Muscle Nerve. 2003;27(2):180-187.

4. Barisic N, Müller J S, Paucic-Kirincic E, Gazdik M, Lah-Tomulic K, Pertl A, et al. Clinical variability of CMS-EA (congenital myasthenic syndrome with episodic apnea) due to identical CHAT mutations in two infants. European Journal of Paediatric Neurology. 2005;9(1):7-12.

5. Mallory LA, Shaw JG, Burgess SL, Estrella E, Nurko S, Burpee TM, et al. A case of congenital myasthenic syndrome with episodic apnea. Pediatric Neurology. 2009;41(1):42-45.

6. Schara U, Christen H-Jr, Durmus H, Hietala M, Krabetz K, Rodolico C, et al. Long-term follow-up in patients with congenital myasthenic syndrome due to CHAT mutations. European Journal of Paediatric Neurology. 2010;14(4):326-333.

7. Yis U, Becker K, Kurul SH, Uyanik G, Bayram E, Haliloğlu G, et al. Genetic Landscape of Congenital Myasthenic Syndromes From Turkey: Novel Mutations and Clinical Insights. Journal of Child Neurology. 2017;32(8):759–765.

8. Shen XM, Crawford TO, Brengman J, Acsadi G, Iannaconne S, Karaca E, et al. Functional consequences and structural interpretation of mutations of human choline acetyltransferase. Hum Mutat. 2011;32(11):1259-1267.

9. Yeung WL, Lam CW, Fung LW, Hon KL, Ng PC. Severe congenital myasthenia gravis of the presynaptic type with choline acetyltransferase mutation in a Chinese infant with respiratory failure. Neonatology. 2009;95(2):183-186.

10. Arredondo J, Lara M, Gospe SdM, Mazia CG, Vaccarezza M, Garcia Erro M, et al. Choline Acetyltransferase Mutations Causing Congenital Myasthenic Syndrome: Molecular Findings and Genotype-Phenotype Correlations. Hum Mutat. 2015;36(9):881 - 893.

11. Tan J-S, Ambang T, Ahmad-Annuar A, Rajahram GS, Wong KT, Goh KJ. Congenital myasthenic syndrome due to novel CHAT mutations in an ethnic kadazandusun family. Muscle & Nerve. 2016;53(5): 822-826.

12. Natera-De BD, Töpf, A, Vilchez JJ, González-Quereda, L, Domínguez-Carral, J, Díaz-Manera, J, et al. Molecular characterization of congenital myasthenic syndromes in Spain. Neuromuscular Disorders Nmd. 2017;27(12) :1087-1098.

13. Schwartz M, Sternberg D, Whalen S, Afenjar A, Isapof A, Chabrol B, et al. How chromosomal deletions can unmask recessive mutations? Deletions in 10q11.2 associated with CHAT or SLC18A3 mutations lead to congenital myasthenic syndrome. American Journal of Medical Genetics Part A. 2018;176(1):151-155.

14. Liu Z M, Fang F, Ding C H, Zhang W H, Deng J, Chen C H,et al. Clinical and genetic characteristics of congenital myasthenia syndrome with episodic apnea caused by CHAT gene mutation: a report of 2 cases. Zhonghua Er Ke Za Zhi. 2018(3):216-220.

15. McMacken G, Whittaker RG, Evangelista T, Abicht A, Dusl M, Lochmüller H. Congenital myasthenic syndrome with episodic apnoea: clinical, neurophysiological and genetic features in the long-term follow-up of 19 patients. Journal of Neurology. 2018;265(1):194-203.

16. Arican P, Gencpinar P, Cavusoglu D, Olgac Dundar N. Clinical and Genetic Features of Congenital Myasthenic Syndromes due to CHAT Mutations: Case Report and Literature Review. Neuropediatrics. 2018;49(4), 283-288.

17. Liu Z M, Zhang L, Shen DM, Ding CH, Yang XY, Zhang WH,et al. CHAT Compound Heterozygous Gene Mutations of a Large Deletion and a Missense Variant in a Chinese Patient With Severe Congenital Myasthenic Syndrome With Episodic Apnea. Front Pharmacol, 2019;10:259.
