# Supplementary material for: Congenital Myasthenic Syndrome Caused by a Novel Hemizygous CHAT Mutation
Source: Front Pediatr. 2020 Apr 28;8:185. doi: 10.3389/fped.2020.00185 (PMC7198756; doi:10.3389/fped.2020.00185)
Supplement: Supplementary file 2 [file Table_2.DOCX]

Table 2. The copy number variation of the 4.9 Mb deletion in this study and its highly overlapping deletion fragment are pathogenic or suspected to be pathogenic.

| Decipher ID | Fragment(chr10) | Assessment | Clinical feature |
| --- | --- | --- | --- |
| 282374 | 47011584-51664079 | pathogenic | medium mental retardation |
| 327088 | 46287821-51861466 | suspected pathogenic | ears forward, and protruding ears, congenital nystagmus, unilateral Microphthalmos |
| 351980 | 46984913-51804163 | suspected pathogenic | mental retardation |
| -  (Our patient) | 46123781-51028772 | pathogenic | apnea, dyspnea, cyanosis, ventilator dependence, high carbon dioxide pressure, hypotonia, convulsions |
